# Gastric Emptying and Its Correlation With Weight Loss and Body Mass Index in Patients With an Intragastric Balloon: A Prospective Study With Six Years of Follow-Up

**DOI:** 10.7759/cureus.32599

**Published:** 2022-12-16

**Authors:** Sérgio Barrichello, Igor Braga Ribeiro, Thiago F de Souza, Manoel dos Passos Galvão Neto, Eduardo Grecco, Jaques Waisberg

**Affiliations:** 1 Department of Gastrointestinal Surgery, Faculdade de Medicina do ABC (FMABC), Santo André, BRA; 2 Serviço de Endoscopia Gastrointestinal do Departamento de Gastroenterologia, Hospital das Clínicas da Faculdade de Medicina da Universidade de São Paulo, Sao Paulo, BRA

**Keywords:** intragastric balloon, obesity, weight loss, gastric emptying, gastric balloon

## Abstract

Background

Obesity is the most well-established and prolonged pandemic in modern society. Having a better understanding of the available tools is important to improve weight loss and make the strategies more productive. This study aims to evaluate the effect of intragastric balloon (IGB) on gastric emptying time, its relationship with weight loss after IGB removal, and weight maintenance after six years.

Methodology

This prospective study analyzed data from patients undergoing IGB placement. A six-years follow-up was performed and data about weight maintenance were collected. We analyzed the impact of the IGB on gastrointestinal motility and its correlation with weight loss.

Results

Of the 20 patients included in the study, 52.4% were diagnosed with class I obesity and 47.6% with class II obesity. The mean weight of the patients was 96.5 ± 11.9 kg at baseline, 79.6 ± 11.4 kg at the time of IGB removal, 81.8 ± 9.1 kg at six months, and 93.2 ± 14.3 kg six years after IGB removal. The mean difference between the initial weight and that measured immediately after IGB removal was 16.68 ± 5.71 kg. Regarding gastric emptying time, there was a difference in retention on comparing the measurement before balloon placement to that after the balloon was in place (72.9% vs. 86.8%) after one hour of food intake. Comparing two hours after food intake, patients before IGB placement had a 30.6% food retention, while patients with IGB in place had a 69.2% retention.

Conclusions

In patients with class I or II obesity, the use of an IGB delayed gastric emptying of foods but showed no direct correlation with weight loss. Weight loss achieved after IGB placement was maintained in half of the patients at a six-year follow-up.

## Introduction

Bariatric and metabolic surgery is the gold standard for the treatment of moderate and severe obesity, whereas the clinical approach is successful in treating overweight patients. However, in several patients, neither option is appropriate [1-3]. In recent years, endoscopic bariatric and metabolic therapies have been developed to fill the gap between surgical treatment and clinical/behavioral approaches [4-6]. In addition to their minimal invasiveness, the efficacy and safety of endoscopic procedures make bariatric endoscopy a good strategy for the initial management of obesity and being overweight [7,8].

Intragastric balloons (IGBs), which were initially introduced in 1985, are now widely used in the treatment of obesity [4]. An IGB is an elastic silicone device filled with 400-700 mL of saline solution and introduced under endoscopic vision. As a weight loss aid, it is more efficient than dietary therapies alone [9,10]. The principle of this technology is that the IGB occupies space within the stomach, resulting in a sensation of early satiety. Although the mechanism of action remains poorly understood, it appears to be related to hormonal changes and changes in gastrointestinal motility [11-13].

Contact between the IGB and the gastric fundus has been considered to be partially responsible for the success of the treatment. This theory is supported by the fact that such contact would promote changes in the hormonal component of satiety due to gastric distention [14]. However, studies by Mathus-Vliegen et al. [15] and Fuller et al. [16] contradict this hypothesis. The relationship between gastric emptying time and the sensation of satiety has been proven [17], along with the relationship between the presence of an IGB and increased food retention [12]. It remains unclear whether there is a direct relationship between the use of an IGB and weight loss, as well as when the IGB-induced decrease in gastric emptying speed would have the greatest impact on the treatment outcomes.

There is controversy regarding the importance of hormones in the mechanism of action of IGBs mainly in relation to ghrelin [14,15,18]. In addition, gastrointestinal motility is the most important factor related to the efficacy of IGB use [19,20]. Therefore, a better understanding of the changes in the pattern of food retention is essential to optimizing multidisciplinary strategies in the approach toward obesity treatment.

Since 1966, gastric emptying scintigraphy (GES) has become the standard approach to measure gastric motility in clinical practice because it provides a physiologic, non-invasive, and quantitative measurement of gastric emptying [21]. Thus, in the present study, we employed gastric scintigraphy to investigate the relationship between the presence of an IGB and the speed of gastric emptying of solid and liquid foods in the first and second hours after ingestion of a test meal and whether this is related to weight loss. In addition, we analyzed weight pre-procedure, immediately after device removal, six months later, and six years later.

## Materials and methods

Study design

This prospective study included a total of 20 patients who underwent IGB placement in 2015. In all patients, we analyzed the impact of the gastric balloon on gastrointestinal motility and its correlation with weight loss. We performed analyses related to pre-procedure weight, immediately after device removal, six months later, and six years later.

A loss equal to or greater than 10% of total weight loss (TWL) or 25% of the percent excess weight loss (%EWL) was considered successful treatment after device removal within six months and after six years of follow-up (maintenance of weight loss).

This study was conducted in a tertiary hospital. Ethical approval for the study was obtained from the Research Ethics Committee of the Faculdade de Medicina do ABC (reference number: 2.102.018; CAE protocol number: 67123717.6.0000.0082). Written informed consent was obtained from all patients within the validation dataset collected in our hospital.

Patient selection

We recruited patients who did not, based on body mass index (BMI), meet the criteria for bariatric surgery. The inclusion criteria were being ≥18 years of age, having a BMI of 30-40 kg/m^2^, and having previously made unsuccessful attempts at weight loss with diet and exercise. The exclusion criteria were patients with hiatal hernia larger than 5 cm, patients with gastrointestinal ulcers, pregnant women, patients using proton pump inhibitors (PPIs) or anticoagulants, those with motility alterations, those with type 2 diabetes mellitus, or those with a history of upper gastrointestinal tract surgery.

Intragastric balloon placement and removal

Each IGB was inserted under intravenous sedation. The IGB employed (Corporea; Medicone Corporea, Cachoeirinha, Brazil) is a single silicone device similar to the Orbera IGB manufactured by Apollo Endosurgery (Austin, TX, USA).

The IGB was inserted orally with the patient under sedation. Passing through the esophagus the balloon was placed in the gastric cavity under endoscopic view. Laid over the great curvature the IGB was filled with 600 cc of saline solution with 10 cc of methylene blue. The catheter and the endoscope were removed after the balloon was filled, and the balloon was left freely in the stomach. Patients were advised to follow a specific diet. During the first two weeks, they were allowed to consume a clear liquid diet that advanced to a more regular diet after two weeks depending on the patient’s complaints. Analgesics (paracetamol 500 mg every eight hours) and antiemetics (dimedihydrate 25 mg every eight hours) were prescribed for the first five days and PPIs (omeprazole 40 mg per day) were to be taken during the treatment.

After the IGB had been in the stomach of the patient for approximately six months, it was removed under general anesthesia. The IGB was punctured and drained endoscopically. After the balloon was deflated, it was retrieved through the mouth.

Weight loss assessment

The body weight and height of the patients were obtained before the balloon placement. We evaluated weight loss and BMI immediately after device retrieval (in six months), six months later, and six years later. BMI was calculated using the following formula: weight/height^2^.

The %TWL was calculated using the following formula: %TWL = [(initial weight) - (postoperative weight)]/[(initial weight)]/100.

The percentage excess BMI loss (%EBMIL) was calculated using the following formula: %EBMIL = [ΔBMI/(initial BMI - 25)]/100.

The %EWL was calculated using the following formula: %EWL = [(initial weight) - (postoperative weight)]/[(initial weight) - (ideal weight)]. Ideal weight was defined by the weight corresponding to a BMI of 25 kg/m^2^.

Gastric emptying

Gastric emptying was evaluated 15-30 days before IGB placement and 8-12 weeks after the placement. Scintigraphy with technetium-99m was employed to quantify gastric emptying of liquid and solid foods over a two-hour period. Scintigraphy is recommended by the American Neurogastroenterology and Motility Society to measure the rate of gastric emptying. The test meal consisted of an omelet made with three medium-sized chicken eggs, prepared with a mixture of colloidal sulfur marked with 185 MBq of technetium-99m. Participants were studied in the morning after fasting for at least 10 hours. The test meal was consumed in five minutes and was followed by the ingestion of 25 mL of water. From time zero, immediately after eating the meal, and every 10 minutes, a 60-second image was obtained, until 120 minutes were completed. Images were taken one-minute anterior and one-minute posterior with the patient in front of a gamma camera. Medications that could affect gastric emptying, such as narcotics, anticholinergic drugs, and prokinetic agents, were discontinued five days before the test.

Follow-up

During the initial six-month period with the IGB, patients were followed up with monthly consultations. After removing the IGB, patients returned to their daily routines with recommendations for diet and exercise. Six months and six years after device removal, all patients were recalled for new weight analysis.

Statistical analysis

The Friedman test was used to compare weight and BMI values. The Wilcoxon paired t-test was used to evaluate statistically significant differences in retention after one hour, retention after two hours, and time to empty. Pearson correlation analyses (r^2^) were used to assess the linear association between weight loss and change in gastric emptying time and between BMI loss and gastric emptying time after the procedure. To verify the influence of each variable on retention and emptying time, we used multiple regression analyses. R software (R Foundation for Computer Statistics, Vienna, Austria) was used for data analysis. A two-tailed p-value of <0.05 was considered statistically significant. Data are presented as mean ± standard deviation (SD).

## Results

A total of 20 patients were enrolled in the study, with a mean age of 34.19 ± 6.16 years (range = 23-48 years). Of the 20 patients evaluated, one (5%) was infected with *Helicobacter pylori*, four (20%) had dyslipidemia, and two (10%) had been diagnosed with depression. During the first three days after IGB placement, 12 (60%) patients complained of nausea and vomiting, but only one was readmitted for intravenous hydration. No early IGB removal was necessary. Obesity was categorized as class I in 11 (55%) patients and as class II in nine (45%) patients (Table 1).

**Table 1:** Characteristics of the study population. SD = standard deviation; BMI = body mass index

Parameters	n	%
Sex		
Female	19	95%
Male	1	5%
Age (mean ± SD)	34.6 ± 6.6	
BMI classification		
Obesity Grade 1	11	55.00
Obesity Grade 2	9	45.00

The mean weight of the patients was 96.5 ± 9.9 kg at baseline, 79.6 ± 9.1 kg at the time of IGB removal, 81.8 ± 9.1 kg at six months, and 93.2 ± 14.3 kg six years after IGB removal (Table 2).

**Table 2:** Weight and BMI of patients with class I or II obesity treated with an IGB, before its placement, immediately after its removal, and six months and six years after its removal. SD = standard deviation; CI = confidence interval; IGB = intragastric balloon; BMI = body mass index

Time point	Mean	SD	Minimum	Maximum	95% CI
Before IGB placement					
Weight (kg)	96.53	9.97	74.50	126.00	88.90-98.52
BMI (kg/m²)	35.17	2.32	30.00	39.7	33.55-35.78
At IBG removal					
Weight (kg)	79.65	9.13	59.00	105.00	72.41-81.21
BMI (kg/m²)	29	2.43	22.20	34.08	27.24-29.59
Six months after IGB removal					
Weight (kg)	81.88	9.13	61.00	106.00	72.41-81.21
BMI (kg/m²)	29.79	2.81	25.90	36.20	27.98-30.69
Six years after IGB removal					
Weight (kg)	93.2	14.3	72.5	124	85.44-96.32
BMI (kg/m²)	34	4.3	28	44	31.98-38.92

The mean percentage of excess body weight loss was greater than 50% immediately after IGB removal and after six months of follow-up. After IGB removal, 95% of the patients were found to have lost more than 10% of their TWL and 25% of their excess body weight. Six months after IGB removal, two (10%) patients had gained weight. Six years after removal, two patients were lost to follow-up as they were referred for bariatric surgery. Of the remaining 18 patients, seven (38.8%) lost 10% or more of total body weight and maintained the weight loss.

As shown in Table 3, there was a significant decrease in the mean BMI after IGB treatment (6.17 ± 1.93 kg/m^2^; p < 0.01). The mean BMI was 29 ± 2.43 kg/m^2^ at the time of IGB removal and 34 ± 4.3 kg/m^2^ after six years.

**Table 3:** Summary of results of patients before using the IGB, immediately after placement, and six years after removal. Change in BMI (ΔBMI): ΔBMI = (initial BMI) - (postoperative BMI). Percentage of total weight loss (%TWL): %TWL = [(initial weight) - (postoperative weight)]/[(initial weight)]/100. Percentage excess BMI loss (%EBMIL): %EBMIL = [ΔBMI/(initial BMI - 25)] 100. Percentage excess weight loss (%EWL): %EWL = [(initial weight) - (postoperative weight)]/[(initial weight) - (ideal weight)]. Ideal weight was defined by the weight corresponding to a BMI of 25 kg/m^2^. *: Immediately after removal of the intragastric balloon. BMI = body mass index; SD = standard deviation

Patient	Initial BMI (kg/m^2^)	Final BMI* (kg/m^2^)	ΔBMI (kg/m^2^)	Initial weight (kg)	Final weight* (kg)	%TWL	%EWL	Weight six years (kg)	%EWL six years	%TWL six years	BMI six years
1	35.56	30.12	5.44	98	83	15.31	0.52	78.00	0.69	20.41	28.31
2	33.86	26.26	7.6	96.7	75	22.44	0.86	95.00	0.07	1.76	33.26
3	33.72	27.89	5.84	90.7	75	17.31	0.67	82.00	0.37	9.59	34.49
4	33.87	22.21	11.67	90	59	34.44	1.31	81.00	0.38	10.00	30.49
5	32.51	28.41	4.1	103	90	12.62	0.55	115.00	-0.5	-11.65	36.30
6	38.05	31.24	6.81	95	78	17.89	0.52	72.50	0.69	23.68	29.04
7	34.66	27.31	7.35	99	78	21.21	0.76	108.00	-0.33	-9.09	37.81
8	39.26	32.85	6.41	98	82	16.33	0.45	75.00	0.65	23.47	30.04
9	37.83	29.38	8.45	103	80	22.33	0.66	85.00	0.52	17.48	31.22
10	39.77	33.14	6.63	126	105	16.67	0.45	124.00	0.04	1.59	39.14
11	34.41	28.73	5.68	103	86	16.5	0.6	92.00	0.39	10.68	30.70
12	37.29	29.4	7.89	104	82	21.15	0.64	93.70	0.3	9.90	33.60
13	37.39	32.05	5.34	91	78	14.29	0.43	108.00	-0.56	-18.68	44.38
14	30.02	25.34	4.68	93	78.5	15.59	0.93	89.00	0.26	4.30	28.73
15	39.79	34.08	5.71	115	98.5	14.35	0.39	97.00	0.42	15.65	33.56
16	32.46	25.97	6.49	75	60	20	0.87	-	-	-	-
17	35.12	30.44	4.69	102.7	89	13.34	0.46	106.00	-0.11	-3.21	36.25
18	33.09	30.86	2.23	89	83	6.74	0.28	-	-	-	-
19	31.83	26.91	4.91	74.5	63	15.44	0.72	84.00	-0.59	-12.75	35.88
20	32.81	27.34	5.47	84	70	16.67	0.7	93.00	-0.45	-10.71	36.33
Mean	35.17	29	6.17	96.53	79.65	17.53	0.64	93.23	0.12	4.58	33.72
SD	2.83	2.97	1.93	11.97	11.48	5.44	0.24	14.31	0.44	13.25	4.20

After IGB removal, all but one patient with class I obesity was recategorized as being overweight or even of normal weight, and all patients with class II obesity were recategorized as having class I obesity.

Regarding the time of gastric emptying, it was noted that patients without IGB showed retention of 72.9% of food one hour after ingestion. In patients with IGB, retention was 86.2% (Table 4). Gastric retention one hour after ingestion in patients without IGB was very similar compared to patients with IGB two hours after ingestion, with retention of 72.9% and 69.2%, respectively (Table 4).

**Table 4:** Mean gastric retention of patients without the use of IGB and with the use of IGB after one and two hours of food intake. ^a^: Wilcoxon test. IGB = intragastric balloon; SD = standard deviation; CI = confidence interval

Study	Mean (%)	SD (%)	Median (%)	Minimum (%)	Maximum (%)	95% CI	P-value^a^
One hour							
Pre-IGB	72.9	14.12	68.55	48.80	100.00	66.46-80.34	0.02
Post-IGB	86.2	21.89	88.50	60.20	100.00	78.21-91.59	
Two hours	28.1	16.99	29.25	12.00	81.70	22.82-39.72	
Pre-IGB	30.6	16.99	29.25	12.00	81.70	22.82-39.72	
Post-IGB	69.2	29.32	67.30	21.00	100.00	51.33-80.50	<0.01

Table 5 presents the data on weight regain noted in the patients.

**Table 5:** Analysis of weight regain at six months and six years. The average weight gain observed in the period was higher after six years of the procedure. SD = standard deviation

	Six months	Six years
No regain	38.39%	22.22%
0.1–50%	50%	16.67%
50.01–99.99%	11.12%	27.78%
>100%	0.00%	33.33%
Weight regain (kg)	Mean	SD
Six months	1.9	4.2
Six years	12.7	12.1
P-value	0.003	

Patients were analyzed at six months and six years after IGB removal. It was found that about 11% of patients regained more than 50% of the weight lost at the end of six months; however, at the end of six years, the percentage was about 61.11%. At the end of six years, 50% of the sample maintained at least 25% EWL, and 66.67% of patients had sustained some percentage of their weight loss.

Multiple linear regression

We verified whether the variable emptying time after the procedure influenced the weight (in kg) lost by patients (Table 6).

**Table 6:** Correlation between the weight lost (in kg) after the procedure and emptying time after the procedure.

Total number of patients (N)	20
Pearson’s correlation	0.39
P-value	0.08

There was no evidence of a significant correlation between weight (in kg) lost after the procedure and emptying time after the procedure.

We observed that the correlation between the BMI lost after the procedure and emptying time had a positive but not significant correlation (Table 7).

**Table 7:** Correlation analysis between pounds lost after the procedure and emptying time after the procedure.

Total number of patients (N)	20
Pearson’s correlation	0.42
P-value	0.07

On adjusting a linear regression model to explain the influence of emptying time after the procedure on lost BMI and weight after the procedure, we observed that both models were not significant for weight loss (p = 0.08) and BMI loss (p = 0.07). In the weight loss model after the procedure, the emptying time explained only 15.9% of the variation in the response explained by the model. The model for the loss of BMI after the procedure, however, explained only 17.4% of the variation in the response explained by the model. However, both models did not show statistical significance.

## Discussion

Gastrointestinal motility and the neurohormonal effects of digestion are closely linked to the excitation or inhibition responses of the branches of the vagus nerve. The vagus nerve acts through afferent neurons that express chemoreceptors and mechanoreceptors, which, in turn, signal the sensation of satiety to the nucleus of the solitary tract in the rhombencephalon [22]. Therefore, vagal activity is mediated by gastric distention, which has been shown to be related to a sensation of satiety and decreased food intake due to the alteration in gastric emptying [12,23].

Since the advent of IGBs, various hypotheses have been proposed to explain the mechanism of action of these devices, including the placebo effect, hormonal changes, lifestyle changes, neuronal activity, and altered gastrointestinal motility [24]. One of the determinants of the outcome of endoscopic IGB placement is the alteration of gastrointestinal motility because the speed of gastric emptying is considered a major determinant of the sensations of satiety and fullness [12,25].

Scintigraphic quantification of gastric emptying is the most appropriate method for evaluating gastrointestinal motility-related diseases [21,26]. In this study, scintigraphy was used to measure first- and second-hour gastric emptying before and after IGB placement, according to the short validation protocol proposed by Pelletier et al. [27], which promotes greater adherence and better acceptance by patients [12].

In our study, there was a significant change in gastric emptying time after IGB placement. Through the use of scintigraphy, the gold standard for investigating gastric emptying, the gastric emptying time for foods was found to be higher after IGB placement. The average retention of the test meal in the first hour was 72.9% and 86.8% before and after IGB placement, respectively. Other studies that used scintigraphy as the method of choice for measuring gastric emptying after IGB placement also reported a significant increase in gastric emptying time [12,20].

Although there is sufficient data in the literature regarding the results of gastric emptying in patients treated with an IGB, there is little information on the gastric emptying rate in the first and second hours after food intake. In this study, IGB placement demonstrated an increase in the rate of food retention in the second hour after ingestion of the test food at 69.2%, which was more than twice compared to patients without the use of IGB. Similar results were reported by Gomez et al. [12]. The mean retention of the test meal in the first hour without IGB was very similar to the mean retention in the second hour with IGB at 72.9% and 69.2%, respectively. Decreasing the speed of gastric emptying in the first and second hour after a meal promotes the sensation of gastric fullness, thus facilitating adjustment of the ingested volumes and the consequent weight loss, in which other variables, such as hormonal changes, changes in lifestyle, and diet modification, also participate.

The impact that hormonal changes have on the response to IGB treatment is yet to be fully elucidated. Mathus-Vlieglen et al. [15] observed decreased secretion of cholecystokinin and pancreatic polypeptide after IGB placement, suggesting that these reductions were related to the increase in gastric emptying time, although not to weight loss or the sensation of satiety. Mion et al. [18] observed decreased plasma levels of ghrelin and reduced gastric emptying after IGB placement, and the weight loss achieved was attributed to the lower ghrelin levels rather than to the reduction in gastric emptying. In contrast, another study showed that changes in preprandial and postprandial ghrelin levels in IGB patients were not related to the sensation of satiety [28].

In our study, no serious complications were identified, underscoring the fact that the use of an IGB is safe [29-34]. The mean weight loss, quantified immediately after IGB removal, was 16.8 kg, similar to that reported in other studies in which an IGB was left in place for six months [35,36]. After the placement of the IGB and the dietary orientation, the mean change in BMI was 6.1 kg/m^2^, and the percentage of excess body weight loss was greater than 50%, values comparable to those reported by other authors [37,38]. On follow-up after six years of the procedures, 50% of the patients maintained a loss of %EWL greater than 25%, which corroborates with the study by Kotzampassi et al. [39], which demonstrated, in about 23% of the patients, maintenance of the loss of the %EWL greater than 20% in five years of follow-up. Ashrafian et al. [40], in a study with morbidly obese patients, reported a five-year drop in BMI of 3.8 kg/m^2^. In our study, after six years, we identified a drop in BMI of 1.79 kg/m^2^.

Although our sample of patients who underwent scintigraphy before and after IGB implantation is the largest in the literature, our study has some limitations. The first is that this study was a prospective study with a sample of a few patients. It is also known that long-term follow-up can be biased as weight maintenance depends on multiple variables such as calories and exercise. We also mention, as a possible bias, the dedication of each patient to remain in the treatment because even those with the use of a restrictive device, such as the IGB, it is possible to increase the number of calories ingested.

## Conclusions

In our patients with class I or II obesity, the use of an IGB delayed gastric emptying of foods but showed no direct correlation with weight loss. Success in weight loss associated with the IGB was maintained in half of the patients at a six-year follow-up.
